# Thymol: properties, synthesis, mechanism of action, and applications

**DOI:** 10.3389/fnut.2026.1774718

**Published:** 2026-03-20

**Authors:** Shizhuo Liu, Junyuan Lei, Hongyang Liu, Tongyu Chen, Chunyan Gao, Dieyu Luo, Qiannan Liao, Xuanrui Liu, Jian Chen, Maxime Bonnave, Francois Serneels, Xiumei Luo, Gengxin Xie, Pan Dong

**Affiliations:** 1 School of Life Sciences, Chongqing University, Chongqing, China; 2 Chongqing No. 1 Secondary School, Chongqing, China; 3 Center for Agriculture and Agro-industry of Hainaut Province, Ath, Belgium; 4 Institute of Urban Agriculture, Chinese Academy of Agricultural Sciences, Chengdu, China; 5 Center of Space Exploration, Ministry of Education, Chongqing, China; 6 College of Environment and Ecology, Chongqing University, Chongqing, China

**Keywords:** action mechanism, bioactivity, food applications, natural compounds, plant essential oil, thymol

## Abstract

Thyme (*Thymus vulgaris*) has long been valued as both a culinary herb and a medicinal plant. Among its constituents, thymol—a key monoterpenic phenolic compound—exhibits a wide range of biological activities, including antimicrobial, antioxidant, anti-inflammatory, and insecticidal properties, which endow it with significant application potential in food-related fields. This review systematically examines the physicochemical properties, biosynthetic pathways, extraction methods, and mechanisms of action of thymol, with a particular emphasis on its prospective applications within the food sector. Traditional extraction techniques such as steam distillation remain in use, whereas emerging methods—including ultrasound-assisted extraction and supercritical fluid extraction—have markedly improved the efficiency, yield, and purity of thymol extraction. From the perspective of synthesis, thymol can be produced chemically via alkylation of *m*-cresol, or it can be generated biologically through the methylerythritol phosphate (MEP) pathway. Thymol exerts its antimicrobial and preservative effects primarily by disrupting microbial cell membranes and inducing oxidative stress, thereby providing the theoretical foundation for its use as a natural food preservative, packaging additive, and functional ingredient. This article further explores the value of thymol in ensuring food quality, enhancing safety control, and extending shelf life. Finally, future research directions are proposed, including optimization of biosynthetic pathways, development of formulations to enhance stability, and exploration of new potential applications at the intersection of antiviral activity, immune modulation, and food-related health.

## 1 Introduction

**Figure 1:**
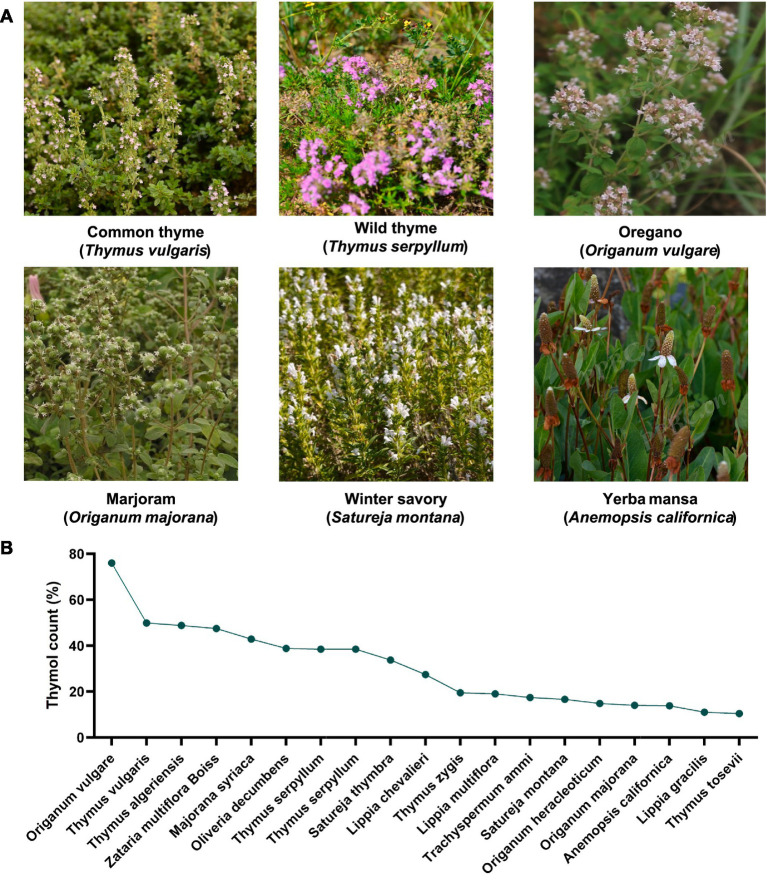
Representative plants containing thymol and the thymol content of different plant species. **(A)** Images of representative plant species containing thymol (Plant Photo Bank of China, https://ppbc.iplant.cn/). **(B)** Comparison of thymol content in essential oils extracted from different plants (5, 6, 125–128).

Thymol (C₁₀H₁₄O), chemically defined as 2-isopropyl-5-methylphenol, is a naturally occurring monoterpenoid phenol that is widely distributed in nature and recognized as one of the principal bioactive components of the essential oil derived from thyme (*Thymus vulgaris* L.) (Figure 1). Thyme was originally discovered in Europe and is now primarily distributed across temperate regions.^1^ It has a long history of application; in ancient times, thyme was employed as an herbal remedy for ailments such as colds and heatstroke (1). Additionally, Europeans have traditionally utilized thyme as a culinary seasoning. Beyond thyme, thymol can be isolated from other plant species (Figure 1A), such as yerba mansa (*Anemopsis californica*), marjoram (*Origanum majorana*), winter savory (*Satureja montana*), wild thyme (*Thymus serpyllum*), oregano (*Origanum vulgare* L.), and glandular thyme (*Thymus glandulosus* L.) (2–4). The level of thymol varies greatly among thymol-containing plants (Figure 1B), ranging from the lowest in *Thymus tosevii* (10.4%) to the highest in *O. yulgure* (76%) (5, 6). The discovery of thymol traces back to the 18th century. In 1719, a German chemist first extracted a pungent-smelling substance from thyme essential oil. During the 1830s, French chemists analyzed the material during their studies of plant essential oils and highlighted its antibacterial properties. By 1853, through the research of Lallemand, the principal component in the essential oil was officially named “thymol” (1).

**Figure 2:**
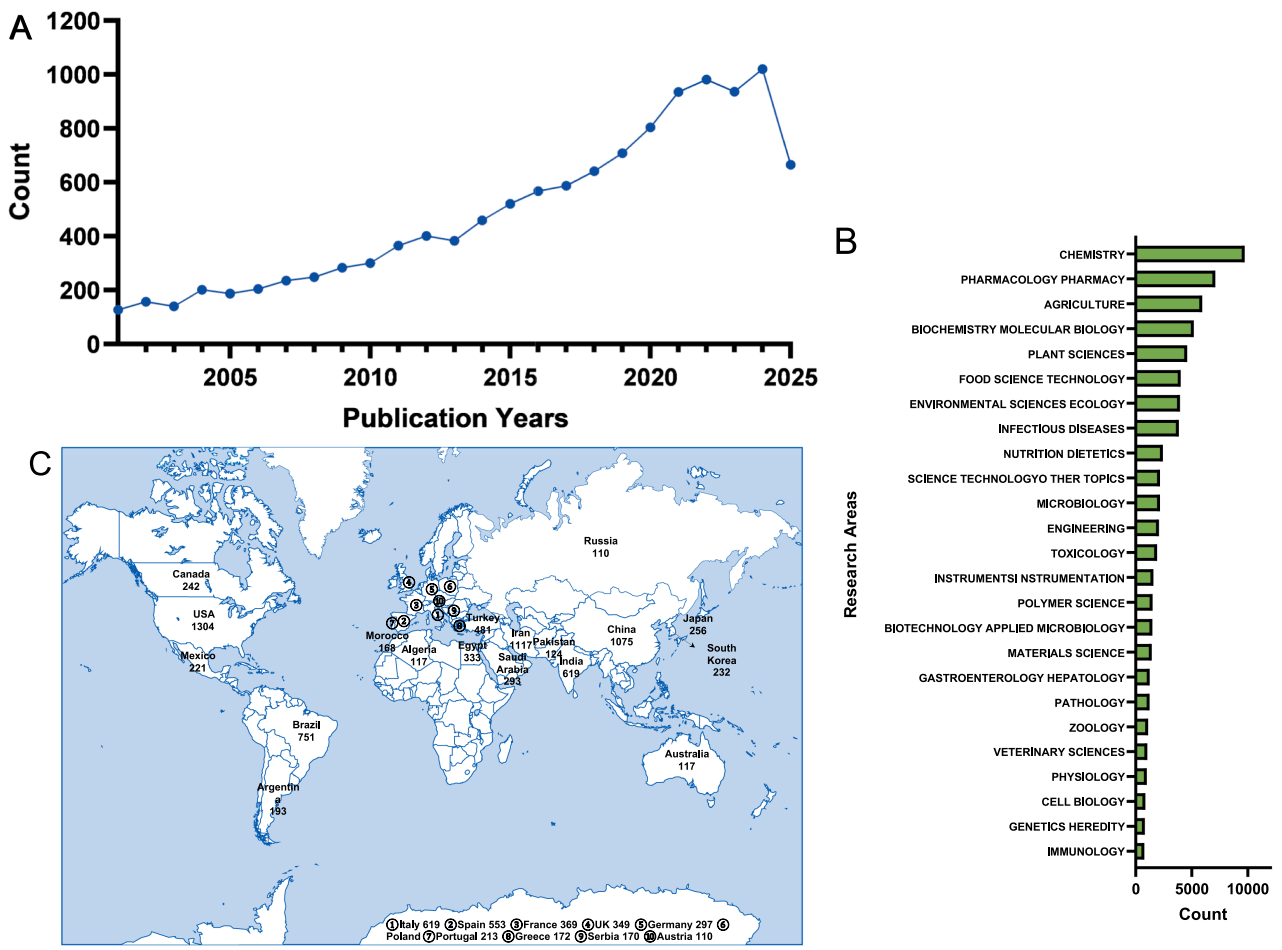
Quantitative analysis of thymol in the Web of Science database (data current as of September 5, 2025). **(A)** Annual publication volume on thymol from 2000 to 2024. **(B)** Top 25 research fields related to thymol. **(C)** Countries/regions with more than 100 thymol-related publications.

In recent years, reports on thymol have grown gradually. Using “thymol” as a keyword, a search of the Web of Science^2^ database indicates that publications related to thymol have risen annually, covering a wide range of fields and countries (Figure 2). The number of articles focusing on thymol has exhibited a marked trend, surging from just over 100 in 2001 to over 1,000 in 2024 (Figure 2A), with the majority of this research originating from the United States, Iran, and China (Figure 2C). As research of thymol continues to deepen, its application potential and significance have become increasingly prominent in considerable relevant fields (Figure 2B). For instance, thymol has received the most extensive attention in the chemical literature, with a total of 9,417 publications, followed by agriculture, pharmacology, and biochemistry and molecular biology. The applications of thymol in the food industry are mainly reflected in the field of preservation. For example, incorporating thymol into edible films (such as starch, gelatin, or chitosan films) allows its slow release on the surface of foods, thereby exerting antimicrobial and antioxidant effects (7). Thymol can also be used as a food additive in meat products to inhibit spoilage (8). In addition, thymol has found certain applications in agriculture, animal husbandry, and other related fields (5, 9). However, despite the increasing number of studies on thymol, existing research still exhibits several limitations: investigations conducted across different disciplines are mostly reported in a fragmented manner, and there is a lack of systematic synthesis regarding its mechanisms of action, application prospects, and limiting factors. Therefore, this review aims to comprehensively summarize recent advances in thymol research, systematically sort out its physicochemical properties, methods of synthesis, biological activities, and application progress, and thereby provide theoretical support for future in-depth studies and industrial-scale utilization.

## 2 Physicochemical properties and derivatives

### 2.1 Physicochemical properties of thymol

Systematic searches for thymol were performed in the SciFinder database^3^ and the American Chemical Society^4^ digital libraries to retrieve a comprehensive summary of its physicochemical properties. Thymol has a molecular formula of C₁₀H₁₄O and a molar mass of 150.22 g/mol. Key attributes include a boiling point of 233 °C, a melting point of 52 °C, a density of 0.9699 g/cm^3^ at 25 °C, a refractive index of approximately 1.522, and a pKa of 10.594 ± 0.10. At room temperature, thymol demonstrates a certain degree of volatility and exists as white crystals with a characteristic aromatic odor similar to thyme, exhibiting sparing solubility in cold water but high solubility in polar solvents (including alcohols, glacial acetic acid, and chloroform), as well as nonpolar solvents such as diethyl ether and benzene (when presenting thymol solubility). Chemically, thymol can be stored stably under dark, cool and sealed conditions; however, it is incompatible with strong acids, strong alkalis, or strong oxidant due to potential reactivity. Although thymol is highly corrosive and slightly toxic, its toxicity is lower than that of phenol. The phenolic hydroxyl group of thymol is its antioxidant active group, which can exert antioxidant effects by scavenging free radicals through dehydrogenation (10). The isopropyl group of thymol enhances its lipid solubility and helps to penetrate the biological membranes (11, 12).

### 2.2 Thymol derivatives

Thymol isomers primarily arise from variations in the position of the hydroxyl group on the phenol ring. Carvacrol, a major isomer of thymol, is naturally present in thyme and represents the most extensively studied compound among all thymol isomers (Supplementary Figure 1), displaying biological activity comparable to those of thymol (13). Using the Mannich reaction, Cui et al. (14) synthesized nine thymol derivatives, which exhibited antibacterial effect against *Escherichia coli*, *Staphylococcus aureus*, and *Salmonella* sp., though slightly less potent than thymol itself. Kaur et al. (15) evaluated the antimicrobial effects of nine different halogenated thymol derivatives and found that 4-chlorothymol demonstrated the strongest efficacy against *S. aureus* and *Staphylococcus epidermidis* and was active against *Candida albicans*, whereas other thymol derivatives have shown no detectable antifungal activity. Moreover, 4-chlorothymol exhibits more than twice the antifungal activity of thymol against *C. albicans*, Chen et al. (16) synthesized 16 novel thioether thymol derivatives and nine of which showed superior inhibitory activity compared to thymol against plant pathogens including *Fusarium oxysporum*, *Cryptococcus mandshurica*, *Rhizoctonia solani*, and *Sclerotinia sclerotiorum*. Through the Morita-Baylis-Hillman reaction, 11 thymol-based adducts were generated. Compound 4 exhibited significantly enhanced anti-*Giardia lamblia* activity, with antigiardial potency 24-fold superior than thymol. Notably, it displayed no cytotoxicity against HEK-293 or HT-29 cells (17).

## 3 Extraction pathway

In addition to *T. vulgaris* L., *C. copticum* L., and *Oliveria decumbens* Vent are commonly utilized for industrial thymol extraction owing to their high thymol contents (18, 19). Traditional methods, such as steam distillation and organic solvent extraction (1), exploit thymol’s volatility and solubility characteristics to isolate it from plants (Table 1). These extraction methods, however, suffer from relatively low efficiency typically less than 0.3%; (1), and encounter significant challenges in separating structurally and chemically similar compounds-especially thymol and carvacrol-due to their analogous physical properties. In addition, organic solvent extraction involves large volumes of petroleum ether or ethanol (20). Modern methods, such as ultrasound-assisted extraction, microwave-assisted extraction, and supercritical fluid extraction, have all been increasingly applied for thymol extraction (Table 1) (21–24). Compared to traditional approaches, these modern techniques enhance extraction efficiency, reduce solvent usage, and shorten extraction time. For instance, ultrasound-assisted extraction (UAE) can improve efficiency by 20–40%, reducing extraction time to under 30 min and solvent consumption by 30–50% (24). Subcritical water extraction (SWE) yields are 2–3 times higher than those obtained by steam distillation, with the entire process typically completed within 20–40 min using only water as the solvent, thereby significantly lowering environmental impact (23). Laboratory studies frequently prioritize green, rapid, and high-recovery extraction strategies, driving the widespread adoption of UAE and SWE, which offer higher selectivity for thymol, greater extraction efficiency, and hold broad application potential. Among these techniques, PLE achieves the highest extraction efficiency (8.32–12.09%), which is higher than that of alternative methods, while yielding thymol concentrations in the range of 5.4–8.9%. Despite its suboptimal extraction efficiency (approximately 0.21%), steam distillation remains the predominant large-scale industrial method for thymol production due to delivering concentrated isolates (36.53% thymol content) and mature process.

**Table 1:** Different thymol extraction methods.

Extraction method	Solvent	Main steps	Essential oil yield	Extraction yield	Reference
Traditional method					
Steam distillation	Distilled water	Grind and maintain a slight boil in distilled water for several hours, then extract with n-hexane	0.21%	36.53%	Xu et al. (20)
Organic solvent extraction	Petrole-um, ethanol	Solid–liquid extraction, filtration, solvent recovery	0.19%	8.73%	Xu et al. (20)
Modern method					
Supercritical CO₂ Extraction	CO₂	High pressure (15–40 MPa) and low temperature (40 °C), extraction for 4 h	3.31–5.25%	16.59–31%	Bermejo et al. (22); Xu et al. (20)
Pressurized liquid extraction	Ethanol, ethyl lactate, Limon-ene	Static extraction for 10 min at 10 MPa and 60–200 ° C	8.32–12.09%	5.4–8.9%	Bermejo et al. (22)
Ultrasound-assisted extraction	Ethanol/water mixture	Ultrasonic disruption of cells to enhance solvent penetration	20–40% higher than conventional solvent extraction	High selectivity for phenols	Yusoff et al. (24)
Subcritical water extraction	Water	High temperature and pressure (100–374 °C, above normal boiling point), water maintained in liquid state	2–3 times higher than steam distillation	Rich in oxygen-containing monoterpenes	Shin and Ko (23)

## 4 Thymol synthesis pathways

During the 20th century, chemists pioneered synthetic methodologies for thymol. Early synthetic routes commonly utilized p-cresol as the starting material, involving a series of chemical transformations, such as alkylation, cyclization, and oxidation, to yield thymol (25, 26). In recent years, investigations into biosynthetic pathways have unveiled novel insights into sustainable thymol production (27, 28).

### 4.1 Chemical synthesis

**Figure 3:**
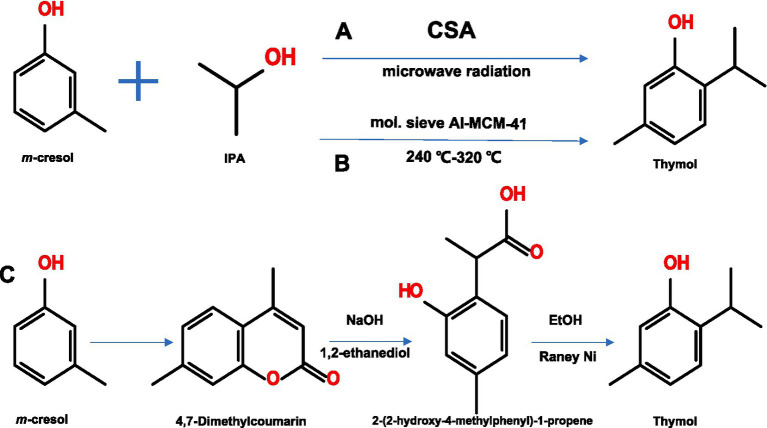
Three chemical synthesis pathways for thymol. IPA: iso-propanol; CSA: carbonized sulfonic acid. **(A–C)** Represent three distinct synthetic routes.

Given its relatively simple structure, the chemical synthesis pathway of thymol is straightforward. As summarized in Figure 3, there are three primary synthetic routes. Under microwave radiation, when the molar ratio of *m-*cresol to iso-propanol (IPA) is 1:5, *m-*cresol can be completely converted into thymol within 4 min using carbonized sulfonic acid (CSA) as a catalyst (29). Notably, both conversion efficiency and reaction rates are remarkably high under these conditions.

The second route employs the same raw materials; however, it utilizes a different catalyst, called molecular sieve (mol. sieve) Al-MCM-41. In the first step, mol. Sieve is uniformly filled in a tubular reactor, which is then heated to 240–320 °C. A raw material mixture (isopropanol: *m-*cresol = (0.7–1.5): 1) is subsequently pumped into the reactor at a space velocity of 0.5–1.5 h^−1^ with a constant peristaltic pump. Following a reaction duration of 10–60 min, the product is collected, extracted with ethyl acetate (EtOAc), dried, and concentrated to obtain thymol with a selective yield exceeding 99% (30).

Thymol can also be synthesized from 4,7-dimethylcoumarin, an intermediate prepared from *m*-cresol (Coumarin intermediate route). The process involves in reacting 4,7-dimethylcoumarin with NaOH in 1,2-ethanediol to generate 2-(2-hydroxy-4-methylphenyl)-1-propene. This intermediate is then hydrogenated in ethanol (EtOH) using Raney nickel (Ni) as a catalyst to create thymol (31). The product purity can reach 98%.

### 4.2 Biosynthesis

**Figure 4:**
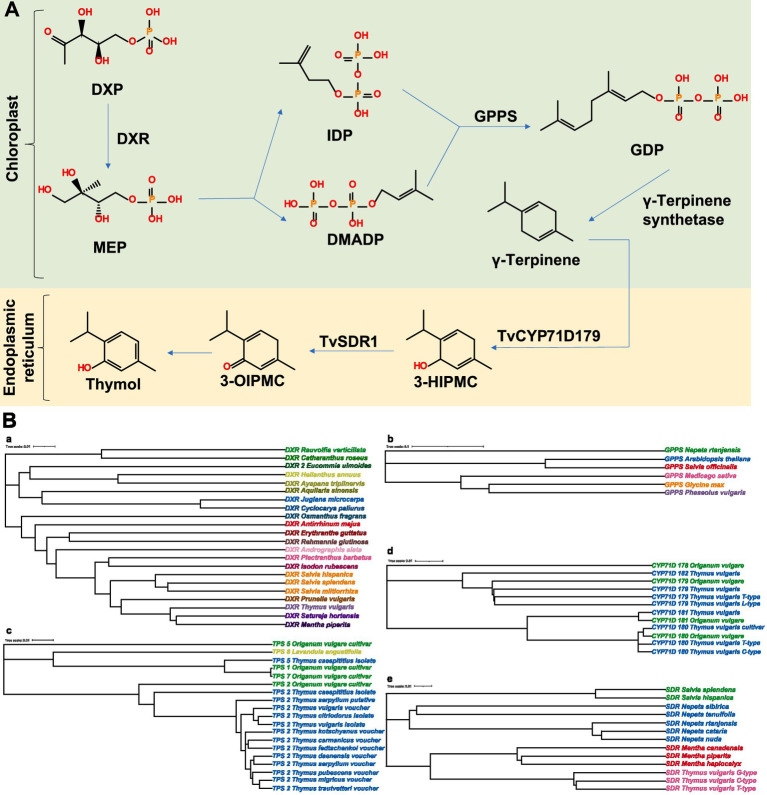
Biosynthesis of thymol. **(A)** Schematic of the thymol biosynthetic pathway. **(B)** Phylogenetic tree of key enzymes involved in the thymol biosynthetic pathway, where each color corresponds a distinct species. a DXR. b GPPS. c *γ*-Terpinene synthetase. d CYP71D. e SDR. DXP: 1-deoxy-D-xylulose-5-phosphate; DXR: 1-Deoxy-D-xylulose-5-phosphate reductoisomerase; MEP: methylerythritol phosphate; IDP: isopentenyl diphosphate; DMADP: dimethylallyl diphosphate; GPPS: geranyl diphosphate synthase; GDP: geranyl diphosphate; SDR: Short-chain dehydrogenase/reductase 3-HIPMC: 3-(3-Hydroxyphenyl)-1-methyl-1H-pyrrolo[2,3-b]pyridine-5-carboxylic acid; 3-OIPMC: 3-(3-Methoxyphenyl)-1-methyl-1H-pyrrolo[2,3-b]pyridine-5-carboxylic acid.

As shown in Figure 4A, the metabolic pathways of thymol and carvacrol have recently been elucidated (27). Thymol biosynthesis involves the coordinated action of a series of enzymes. Specifically, thymol and carvacrol are two phenolic monoterpenes produced via the plastidial methylerythritol phosphate (MEP) pathway, utilizing isopentenyl diphosphate (IDP) and dimethylallyl diphosphate (DMADP) as precursors (32, 33). In the MEP pathway, 1-deoxy-D-xylulose-5-phosphate is irreversibly converted to MEP by 1-deoxy-D-xylulose-5-phosphate reductoisomerase (34). According to Lichtenthaler (35) and Burke et al. (36), geranyl diphosphate synthase (GPPS) is a key enzyme in this biosynthetic pathway, catalyzing the head-to-tail condensation of IDP and DMADP to form geranyl diphosphate (GDP), a universal precursor of monoterpenes. Subsequently, GDP undergoes cyclization catalyzed by *γ*-terpinene synthase, yielding γ-terpinene, an essential intermediate metabolite for thymol and carvacrol biosynthesis (37). γ-Terpinene is hydroxylated at specific positions by cytochrome P450 enzymes belonging to the CYP71D subfamily (e.g., TvCYP71D179). Site-specific hydroxylation of γ-terpinene gives rise to diverse unstable cyclohexadienols; specifically, hydroxylation at the C-3 position yields a crucial precursor for thymol (28, 37). Finally, the cyclohexadienol intermediates are converted into corresponding ketones by short-chain dehydrogenase-reductases (TvSDR1). The resulting ketones undergo keto-enol tautomerization, which facilitates their aromatization into stable phenolic compounds, ultimately forming thymol (28). The gene sequences encoding key biosynthetic enzyme involved in thymol biosynthesis were retrieved from the NCBI database.^5^ A molecular phylogenetic tree was constructed using the Neighbor-Joining method in MEGA^6^ to investigate sequence homology of these enzymes across plant species (Figure 4B). The results reveal that enzymes upstream of the MEP pathway, such as DXR, are generally conserved. GPPS belong to multigene families that show species- and lineage-specific clustering. In contrast, the TPS family has undergone significant expansion in the *Lamiaceae*, particularly in *Thymus* and *Origanum*. For monoterpene terminal modification, CYP71D and SDR play a decisive role in directing precursors toward thymol or carvacrol biosynthesis.

## 5 Biological activity of thymol

### 5.1 Effect on bacteria

#### 5.1.1 Inhibition effect

Numerous studies have demonstrated that thymol and its essential oil possess potent broad-spectrum antibacterial activity, effectively inhibiting the growth of a wide range of pathogenic Gram-negative and Gram-positive bacteria (Table 2). Particularly in the context of the clinically significant “ESKAPE” pathogens, existing literature—though primarily focused on thyme-extracted essential oils rather than isolated thymol and with MIC values still being fully elucidated—indicates a clear inhibitory effect against *Klebsiella pneumoniae* (38). Given its critical relevance to human health, these potential underscores thymol’s strategic value as a natural antimicrobial agent. In addition to *K. pneumoniae*, thymol effectively targets *E. coli* (e.g., O157: H7), *Salmonella*, *Staphylococcus aureus*, and *Mycobacterium tuberculosis* (39–42). Thymol not only reduces bacterial counts but also inhibits and eradicates biofilm formation. Minimum inhibitory concentration (MIC) and minimum bactericidal concentration (MBC) values differ across species, reaching as low as the microgram level, and thymol achieves rapid bactericidal effects within short exposure time (42, 43). Mechanistic studies suggest its actions may involve disrupting cell membrane, interfering with energy metabolism, and inhibiting DNA replication, repair, and transcription, underscoring its potential as a natural antimicrobial agent (44, 45). However, some Gram-negative bacteria (e.g., *Pseudomonas aeruginosa*) exhibit great resistance (39).

**Table 2:** Bacteria inhibited by thymol.

Genus	species	MIC value	Associated diseases	References
*Actinobacillus*	*A. pleuropneumoniae*	31.25 μg/mL	Porcine contagious pleuropneumonia	Wang et al. (129)
*Aeromonas*	*A. hydrophila*	128 μg/mL	Gastroenteritis	Liang et al. (44)
*Bacillus*	*B. licheniformis*		Bovine abortion	Marino et al. (40)
	*B. thuringiensis*			Marino et al. (40)
	*B. subtilis* (MTCC 121)	125 μg/mL		Mathela et al. (41)
*Brucella*	*B. melitensis*	6.25 μg/mL	Brucellosis	Al-Mariri et al. (38)
*Campylobacter*	*C. coli*	1 mM	Campylobacteriosis	Epps et al. (130)
	*C. jejuni*	1 mM	Campylobacteriosis	Epps et al. (130)
*Escherichia*	*E. coli*		Enterohemorrhagic	Marino et al. (40); Abu-Darwish et al. (39)
	*E. coli O157: H7*	1.10 mM	Enterohemorrhagic	Marino et al. (40); Al-Mariri et al. (38)
	*E. coli* (ATCC 15221)	5.0 mg/mL		Moon and Rhee (131)
	*E. coli* (MTCC 723)	250 μg/mL		Trombetta et al. (132); Mathela et al. (41)
*Helicobacter*	*H. pylori*	14.5–58.0 μg/mL	Chronic gastritis	Eftekhar et al. (133)
*Gardnerella*	*G. vaginalis*	97 μg/mL		Braga et al. (134); Al Maqtari et al. (135)
*Klebsiella*	*K. pneumoniae*	3.125 μL/ mL	Pneumonia	Al-Mariri et al. (38)
*Listeria*	*L. innocua*			Marino et al. (40)
	*L. monocytogenes*	0.2 mg/mL	Listeriosis	Pan et al. (42); Moon and Rhee (131)
*Mycobacterium*	*M. bovis*	2.02 μg/mL	Tuberculosis	Andrade-Ochoa et al. (43)
*Proteus*	*P. mirabilis*		Urinary Tract infection	Marino et al. (40)
	*P. vulgaris*		Urinary Tract infection	Marino et al. (40)
	*Proteus* spp.			Marino et al. (40)
*Pseudomonas*	*P. putida*	3.125 μL/mL		Al-Mariri et al. (38)
	*P. fluorescens*	2.0% *w*/*v*		Liu et al. (55)
	*P. aeruginosa*	3.125 μL/mL	Pneumonia	Marino et al. (40)
*Salmonella*	*S. typhimurium*	6.25 μL/mL	Gastroenteritis	Abu-Darwish et al. (39); Al-Mariri et al. (38)
*Serratia*	*S. marcescens*			Marino et al. (40)
*Staphylococcus*	*S. aureus*		Skin and soft tissue infections	Marino et al. (40)
	*S. aureus* (ATCC 68380)	0.31 mg/mL		Marino et al. (40); Nostro et al. (45); Abu-Darwish et al. (39)
	*S. aureus* (MTCC 96)	62.5 μg/mL		Trombetta et al. (132)
	*S. epidermidis*			Mathela et al. (41)
	*S. epidermidis* (MTCC 435)			Nostro et al. (45)
*Streptococcus*	*S. mutans*	125 μg/mL	Dental caries	Mathela et al. (41)
	*S. mutans* (ATCC 25175)	0.5 mg/mL	Dental caries	Lee et al. (136)
	*S. sobrinus*	0.5 mg/mL	Dental caries	Lee et al. (136)
	*S. iniae*	128 μg/mL		Lee et al. (136)
*Yersinia*	*Y. enterocolitica*		Yersiniosis	Liang et al. (44)
	*Y. enterocolitica* (O9)		Yersiniosis	Marino et al. (40)

#### 5.1.2 Mechanism of action

**Figure 5:**
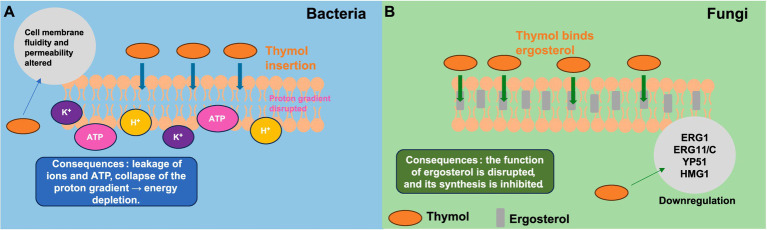
Mechanism of thymol-mediated microbial activity inhibition. **(A)** Thymol disrupts the permeability and fluidity of bacterial cell membranes, resulting in the loss of membrane integrity, impairment of energy metabolism. **(B)** Thymol interferes with fungal growth by affecting the content and function of ergosterol in the fungal cell membrane, ultimately triggering apoptosis. (Icon from https://www.iconfont.cn/).

Transcriptomic analysis of bacteria (*Listeria monocytogenes*, *Streptococcus iniae*) treated or untreated with thymol revealed a large number of significantly differentially expressed genes, which showed substantial enrichment in Gene Ontology (GO) terms, primarily those associated with the bacterial cell membrane (Supplementary Figure 2). For example, thymol exerts significant antibacterial effects against *S. aureus*, primarily by affecting the cell membrane (45, 46). Thymol can integrate into the lipid bilayer of cell membranes, thereby altering their physicochemical properties and perturbing membrane fluidity and permeability (47) (Figure 5A). This property is the basis of its remarkable antibacterial activities. Thymol compromises the barrier, leading to leakage of essential intracellular substances such as potassium ions (K^+^) and adenosine triphosphate (ATP) (Figure 5). The leakage of K^+^ disrupts osmotic balance, whereas the loss of ATP directly depletes bacterial energy supply, ultimately causing cell death (48). Notably, the delocalized electron system (double bonds) and hydroxyl group present in both carvacrol and thymol have been identified as the key structural features influencing their activity (49). These chemical structures enable thymol to act as a proton exchanger, reducing the proton gradient across the cytoplasmic membrane, leading to proton motive force (PMF) collapse and ATP depletion, ultimately resulting in cell death (50, 51).

### 5.2 Effect on fungi

#### 5.2.1 Inhibition effect

Thymol exhibits potent broad-spectrum antifungal activity (Table 3), effectively inhibiting growth and killing diverse clinical and environmental fungi species, including *Candida* spp., *Aspergillus* spp., *Penicillium* spp., *Trichoderma* spp., *Cladosporium* spp., *Alternaria* spp., *Botrytis* spp., *Fusarium* spp., and *Mucor* spp. (52–55). MIC values vary by fungal species, reaching as low as 80–97 μg/mL for some *Candida* species (2), 0.13–0.26 mg/mL for *C. albicans* (56), and averaging 263.3 μg/mL for filamentous fungi (52). In addition, at a concentration of 1 mmol/L, thymol completely inhibit the growth of *Candida lusitaniae* at 25 °C over a 21-d period (54). Inhibitory potency differs among fungal genera, with the strongest activity against *Cladosporium*, followed by *Aspergillus*, *Fusarium*, *Botrytis*, *Penicillium*, *Alternaria*, and *Mucor* (55). Notably, thymol significantly suppresses the growth of *Aspergillus flavus* at a concentration of 350 ppm (57). These findings indicate that thymol has high potential as a broad-spectrum and long-acting natural antifungal agent.

**Table 3:** Fungi inhibited by thymol.

Genus	Species	MIC value	Related diseases	References
*Aspergillus*	*A. fumigatus*	25 ± 2.8 μg/mL	Invasive pulmonary aspergillosis	Dzamic et al. (54)
	*A. flavus*	10 ± 2.8 μg/mL	Invasive aspergillosis	Dzamic et al. (54); Badawy et al. (57)
	*A. niger*	10 ± 2.8 μg/mL		Dzamic et al. (54)
	*A. ochraceus*	10 ± 1.45 μg/mL		Dzamic et al. (54)
*Alternaria*	*A. alternata*	400 μg/mL	Allergic diseases	Abbaszadeh et al. (52)
*Botrytis*	*B. cinerea*	300 μg/mL	Gray mold	Bouchra et al. (2); Abbaszadeh et al. (52)
*Candida*	*C. albicans*	50 ± 2.8 μg/mL	Candidiasis	Braga et al. (134); Dzamic et al. (54); de Castro et al. (53)
	*C. dubliniensis*	0.26 mg/mL		Gallucci et al. (56)
	*C. krusei*	0.13 mg/mL	Candidemia	Gallucci et al. (56); de Castro et al. (53)
	*C. tropicalis*	0.26 mg/mL	Candidemia	Gallucci et al. (56); de Castro et al. (53)
	*C. lusitaniae*			Aznar et al. (137)
*Cladosporium*	*Cladosporium* spp.	100 μg/mL		Abbaszadeh et al. (52)
*Fusarium*	*F. oxysporum*	300 μg/mL	Fusarium wilt	Abbaszadeh et al. (52)
*Penicillium*	*P. citrinum*	250 μg/mL	Citrinin toxicosis	Abbaszadeh et al. (52)
	*P. chrysogenum*	500 μg/mL		Abbaszadeh et al. (52)
	*P. funiculosum*	12.5 μg/mL		Dzamic et al. (54)
	*P. ochrochloron*	25 ± 2.8 μg/mL		Dzamic et al. (54)
*Rhizopus*	*R. oryzae*	450 μg/mL	Mucormycosis	Abbaszadeh et al. (52)
*Saccharomyces*	*S. cerevisiae*			Varga et al. (138)
*Trichoderma*	*T. viride*	10 ± 1.45 μg/mL		Dzamic et al. (54)

#### 5.2.2 Mechanism of action

The antifungal mechanism of thymol shares similarities with that observed in bacteria but exhibits greater complexity (Figure 5B). Among the enriched Gene Ontology (GO) terms, those with the highest degree of overlap were primarily related to the categorys “membrane” and “membrane part” (Supplementary Figure 3). A defining feature of fungal cell membranes is the presence of ergosterol - a unique sterol component that is indispensable for sustaining membrane stability and mediating core membrane functionalities (58, 59). Thymol binds to ergosterol, disrupting its distribution within the membrane, weakening its physical strength, and impairing its permeability barrier function (60, 61). The interaction compromises the structural and functional integrity of the fungal membrane, alters its permeability, and triggers significant electrolyte and small molecule leakage, in turn disrupting normal metabolic processes and ultimately leading to cell death. Thymol can also disrupt ergosterol biosynthesis in fungi, thereby compromising the structural integrity of the fungal cell membrane (60). For example, studies on *Candida* spp. have shown that thymol inhibits growth by targeting the ergosterol pathway (60). Studies across various fungal systems, including *Candida*, *Cryptococcus*, and *Fusarium*, have demonstrated that thymol downregulates key genes in the ergosterol biosynthesis pathway—such as *ERG1*, *ERG11/CYP51*, and *HMG1*—leading to a reduction in intracellular ergosterol levels (62, 63). This disruption impairs the fluidity and permeability of the fungal cell membrane, ultimately inhibiting fungal growth and, in some cases, resulting in cell death. Moreover, in *Cryptococcus neoformans*, this regulation has been linked to the HOG MAPK signaling pathway (62), whereas in *Nakaseomyces glabratus*, thymol affects intracellular ergosterol levels by simultaneously modulating both *ERG1* expression and the HOG pathway (64).

### 5.3 Antioxidant effect and mechanism of thymol

**Figure 6:**
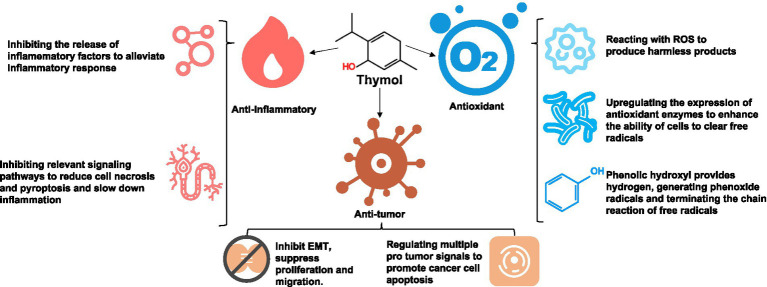
The antioxidant, anti-inflammatory, and anti-tumor activities of thymol (icon from https://www.iconfont.cn/).

Thymol exhibits antioxidant properties through its ability to scavenge excess reactive oxygen species (ROS) to alleviate cellular damage and inflammatory responses (9) (Figure 6). Excessive ROS can lead to lipid peroxidation, protein carbonylation, and DNA oxidative damage, thereby resulting in cell dysfunction and aggravation inflammation. Through its phenolic hydroxyl group, thymol can directly react with ROS, neutralizing them to generate harmless products. For example, Pérez-Rosés et al. (65) demonstrated that thymol reduces ROS production in neutrophils stimulated by phorbol 12-myristate 13-acetate (PMA) and hydrogen peroxide (H₂O₂), while exhibiting myeloperoxidase-inhibitory activity. Thymol has been shown to modulate the expression of antioxidant enzymes both *in vitro* and *in vivo* across various ROS models, primarily by activating the Nrf2–ARE antioxidant response element signaling pathway (66). This system protects cells from oxidative damage by regulating enzyme activity to neutralize free radicals. Specifically, thymol enhances cellular antioxidant capacity by inducing the expression of key antioxidant enzymes, including superoxide dismutase (SOD), catalase (CAT), and glutathione peroxidase (GPx) (67, 68). Via hydrogen donation, the phenolic hydroxyl group reacts with free radicals to form relatively stable phenoxy radicals, thereby terminating the free radical chain reaction and reducing oxidative damage (69).

### 5.4 Anti-inflammatory effect and mechanism of thymol

Studies have shown that thymol inhibits the release of inflammatory factors (70) (Figure 6). For example, a prior study reported that thymol alleviates the inflammatory response in isoproterenol-induced myocardial infarction in rats (*Rattus norvegicus*) by suppressing the release of lysosomal enzymes and downregulating the expression of pro-inflammatory cytokines—tumor necrosis factor (TNF-*α*), interleukin-1β (IL-1β), and IL-6 (71). Additionally, in a lipopolysaccharide-induced inflammation model based on goat (*Capra hircas*) endometrial epithelial cells, thymol significantly reduced the secretion of pro-inflammatory cytokines, thereby mitigating the inflammatory response and constraining inflammatory disease progression (72). Thymol also exerts significant regulatory effects on aberrant cell signaling trigged by exogenous factors. For example, in an *Aspergillus fumigatus*-induced keratitis model, thymol inhibited both cell necrosis and pyroptosis by suppressing the TLR4/MyD88/NF-κB/IL-1β signaling pathway, thereby alleviating its abnormal activation (73). Furthermore, in hepatitis and endotoxin-induced models, thymol has been shown to inhibit the activation of the NLRP3 inflammasome and attenuate inflammation and apoptosis through the AMPK–mTOR–autophagy signaling pathway (74).

### 5.5 Anti-tumor effect and mechanism of thymol

Beyond its redox-regulatory actions, thymol exerts direct antitumor effects across diverse models (Figure 6). In colorectal cancer and other models, thymol has been shown to suppress the phosphorylation of PI3K/AKT and its downstream targets, including mTOR and GSK-3β, accompanied by decreased MMP-2/9 activity and upregulation of E-cadherin (75). These changes collectively weaken cell migration and invasion, and contribute to the reversal of epithelial–mesenchymal transition (EMT). With respect to apoptosis, thymol predominantly activates the mitochondrial pathway, leading to a reduction in mitochondrial membrane potential, release of cytochrome c, activation of caspase-9 and -3, and an increased Bax/Bcl-2 ratio in favor of apoptosis (76–78). This cascade has been consistently validated in cell and mouse models of bladder, breast, and prostate cancers, as well as leukemia and oral cancers (79). Moreover, thymol broadly attenuates growth and survival signaling by remodeling multiple pro-tumor pathways. First, it modulates the MAPK family: in various cancer types, it suppresses the ERK1/2 pathway while activating JNK and p38 to promote apoptosis—effects that align with MMP downregulation (78, 80). Second, thymol interferes with the Wnt/β-catenin and JAK/STAT axes (81, 82). In colorectal cancer, it downregulates β-catenin and inhibits proliferation and migration (82); while in multiple cancer types, it reduces STAT3 phosphorylation and nuclear translocation (82, 83). Third, thymol inhibits NF-κB and downregulates pro-inflammatory cytokines, thereby dampening anti-apoptotic signaling (84).

## 6 Pharmacokinetics

### 6.1 The metabolic pathway of thymol

Following oral administration, thymol is rapidly absorbed in the stomach and the upper segment of the small intestine. Animal studies have demonstrated that thymol is detected in the stomach, intestines, and urine after oral dosing (85, 86). In human studies, healthy volunteers who took a Bronchipret^®^ TP tablet containing 1.08 mg of thymol exhibited detectable plasma metabolites within 20 min, with a time to peak concentration (T_max_) of approximately 1.97 h and an absorption half-life (t_1/2abs_) of only 0.3 h, indicating an extremely absorption rate of rate (87).

After the administration of 50 mg/kg thymol, drug elimination was essentially complete within approximately 24 h (87). Once absorbed, free thymol is rarely present in the bloodstream; instead, it circulates primarily as thymol sulfate, with a peak plasma concentration of roughly 93 ng/mL and a bioavailability of approximately 16% (87). The steady-state volume of distribution is 14.7 L, suggesting that thymol is predominantly distributed in the extracellular fluid (87).

**Figure 7:**
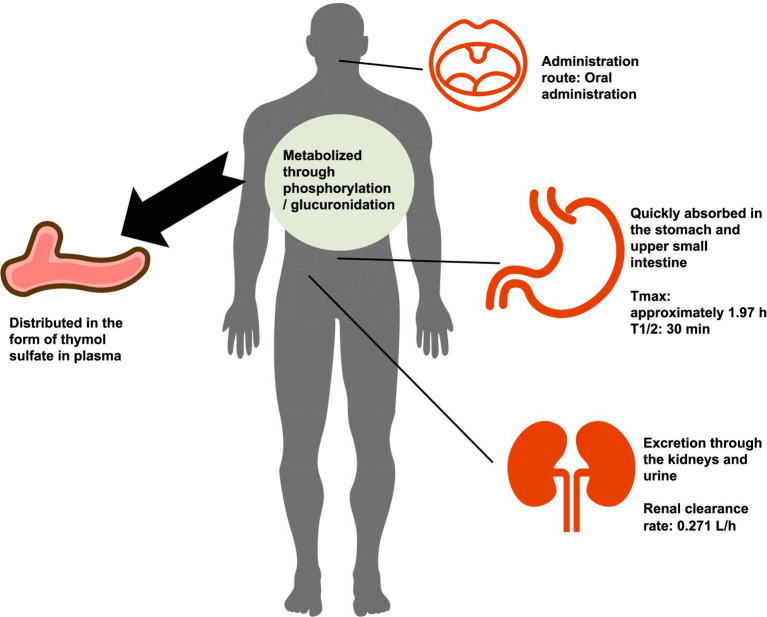
Pharmacokinetics of thymol (Icons from https://www.iconfont.cn/).

In terms of metabolism, thymol undergoes predominant biotransformation via sulfation, and glucuronidation additionally occur as a metabolic route at higher doses, producing key metabolites such as thymol sulfate, thymol glucuronide, and thymol thymohydroquinone sulfate (88–90). These metabolites are mainly excreted via the kidneys (87). Within 6 h post-administration, the major metabolites become detectable in urine, with the excretion profile reaching its peaks within 24 h (87). The combined amount of thymol sulfate and glucuronide in urine accounts for about 16.2% of the administered dose, accompanied by a renal clearance rate of approximately 0.271 L/h (87). Moreover, thymol is rarely identified in feces, indicating that renal excretion is the predominant elimination pathway and that clearance via the kidneys is relatively efficient (86). In summary, thymol exhibits pharmacokinetic characteristics, including rapid absorption, metabolism mainly via sulfation, broad but predominantly extracellular distribution, and primary reliance on renal elimination (Figure 7).

### 6.2 The effect of β-cyclodextrin inclusion on the pharmacokinetic behavior of thymol

In order to optimize its pharmacokinetic characteristics, researchers developed a thymol β-cyclodextrin inclusion complex, supplemented with poly (aminoethyl methacrylate) (DMAEMA) copolymer to mask odors and control volatilization (91, 92).

Following oral administration in a porcine model, a comparative study of free thymol and the sealed-heating (SH EP) formulation reveals distinct kinetic behaviors despite similar peak concentrations (C_max_ of 3.6 ± 0.4 μg/mL for T and 3.8 ± 0.5 μg/mL for SH EP) (92). The most prominent enhancement occurs in the absorption phase: SH EP markedly reduces the time to reach T_max_ from 1.3 h to only 0.5 h. This is further supported by the t_1/2abs_, which drops from 0.9 h to 0.3 h, (92) demonstrating that the inclusion complex acts as superior solubilizer, facilitating immediate drug release in the gastrointestinal environment. The elimination kinetics also exhibit formulation-dependent changes. Although the total bioavailability remains unchanged at approximately 26–27 μg∙h/mL, the elimination half-life (t_1/2 elim_) for SH EP is significantly shorter at 22.3 h compared to 51.9 h for free thymol (92). This suggests a more efficient clearance or altered metabolic rate following complexation. Regarding tissue distribution, while free thymol and SH EP both show minimal accumulation in systemic organs—with concentrations in the liver, kidneys, and lungs ranging from only 37 to 58 ng/g—the SH EP formulation drastically increases intestinal retention. Notably, in the jejunum content, thymol concentration from SH EP reached 333.46 ng/g, nearly five times higher than the 68.91 ng/g found for free thymol (92, 93). Furthermore, the presence of thymol in faeces after 24 h (121.18 ng/g) for the SH EP group, compared to much lower levels in the free drug group, indicates a prolonged gastrointestinal transit time (87, 92, 93). This sustained local presence on the intestinal mucosa, combined with the taste-masking effect of the DMAEMA copolymer (which reduces thymol volatility by over 95%) (92, 93), makes SH EP an ideal delivery system for targeted intestinal antimicrobial therapy. These refined parameters demonstrate that cyclodextrin complexation not only accelerates initial systemic entry but also optimizes the local availability of thymol within the gut.

## 7 Research areas and applications

**Figure 8:**
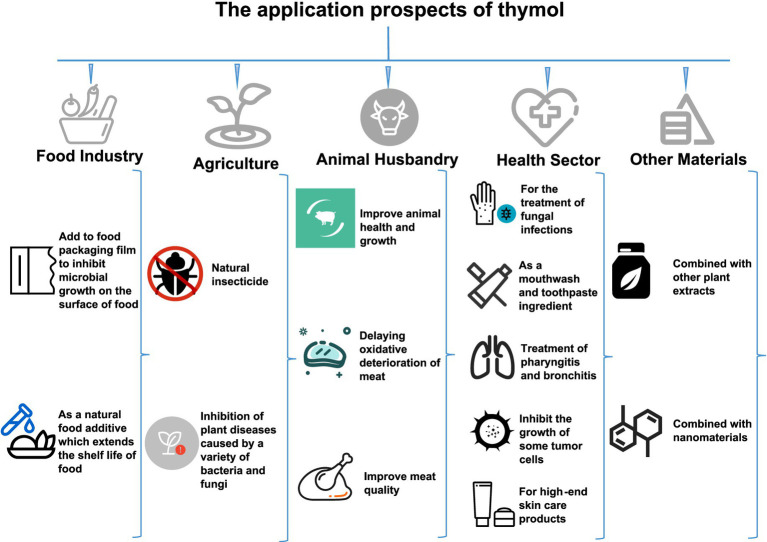
The application prospects of thymol (Icons from https://www.iconfont.cn/).

Owing to its pronounced biological activities, thymol exhibits considerable application potential across multiple fields. It not only meets the growing demand for natural, safe, and environmentally friendly products, but also aligns with the technological development trends in the food and related industries toward diversification and enhancement of functional ingredients (Figure 8). In recent years, the scope of research involving thymol has also expanded progressively.

### 7.1 Applications in food-related fields

#### 7.1.1 Food packing

Thymol can be incorporated into packaging materials as an active antimicrobial component. For example, in the production of meat products, thymol is added to packaging materials to inhibit microbial growth on the product surface, thereby helping to maintain desirable sensory attributes and ensure microbiological safety (94). Thymol can also be used in the packaging of vegetables (such as broccoli and tomatoes). López-Gómez et al. (95) have shown that active packaging containing thymol can significantly reduce ethylene production in vegetables, thereby delaying ripening and extending shelf life. In addition, using thymol-containing packaging films for products such as cheese and milk can suppress the growth of bacteria and fungi within these products (42, 96).

With respect to packaging materials, thymol also exhibits a high degree of adaptability. For instance, the incorporation of thymol into multilayer Ethylene–Vinyl Alcohol Copolymer (EVOH)/Polypropylene (PP) films enable sustained inhibition of common spoilage microorganisms in foods (97). Its diffusion and release behavior can be effectively predicted by modeling, demonstrating the high application potential of thymol in conventional plastic-based active packaging. Thymol can likewise be added to biodegradable polylactic acid (PLA) films, which markedly enhances their antimicrobial and antioxidant properties (98); the controlled-release characteristics of thymol within the film render it suitable for active packaging of high-fat meat products or other highly perishable foods.

#### 7.1.2 Food additive

Thymol can also serve as a green and efficient source of food additives. When incorporated into dairy products such as cheese, milk, and yogurt, thymol can significantly reduce total fungal and bacterial counts during refrigerated storage, while preserving sensory attributes and overall product quality as much as possible (42, 99, 100). For example, Makhal et al. (101) found that the addition of a thymol solution to cheese extended its shelf life from approximately 12 days to 20 days, without exerting any noticeable negative effects on its characteristic flavor. In the context of meat products, thymol can be used as a functional additive during curing (94). The direct incorporation of thymol into marinated beef products significantly inhibits the growth of total viable bacteria and coliforms, delays lipid oxidation, and helps maintain desirable color and sensory quality during chilled storage (102, 103). Although thymol has not yet been directly applied to fruit juices or similar beverages, studies conducted in model juice systems have shown that thymol can markedly reduce the survival of spoilage microorganisms and the production of their metabolic by-products (5). This demonstrates the strong potential of thymol as a natural preservative additive for juice-based products.

#### 7.1.3 Agriculture

In agriculture, thymol finds application in the prevention and management of plant diseases and pests Thymol demonstrates insecticidal activity against insects, rendering it a potential natural insecticide (104). Specifically, it exhibits significant toxicity toward piercing-sucking pests such as whiteflies (*Bemisia tabaci*) and citrus blackflies (*Aleurocanthus spiniferus*) (105). Additionally, thymol can be deployed for the control of bacterial and fungal plant diseases, thereby facilitating the healthy growth of crops. These diseases include grape anthracnose, tea anthracnose, tea ring rot, gray mold, sclerotinia stem rot, mango stem rot, watermelon wilt, wheat fusarium head blight, and rice sheath blight (105–107).

#### 7.1.4 Animal husbandry

The prominent antibiotic properties of thymol underpin its application in the treatment of bacterial diseases in animals, making it a favorable choice as an antibiotic adjuvant in animal husbandry. Thymol is often applied to improve feed conversion rates, enhance poultry immunity through its antibacterial effects, reduce the need for antibiotics, and maintain carcass traits (108). Moreover, thymol has been validated to promote gut health and improve growth performance (109). During the post-production phase, thymol can retard the oxidative spoilage process, which is particularly prevalent in poultry meat (110). Overall, from the perspective of meat quality, the use of plant-based feed additives (PFA) containing thymol and carvacrol yields favorable outcomes, including optimized bacterial loads, improved biochemical characteristics, enhanced meat quality, and modulated fatty acid profile levels in meat products (111).

### 7.2 Applications in cross disciplinary field of food and health

#### 7.2.1 Healthcare sector

Thymol can be incorporated into a range of pharmaceuticals for the treatment of skin infections, where it contributes to of the eradication of pathogenic microbes while concomitantly attenuating skin inflammation (112). For instance, in formulations targeting athlete’s foot and ringworm, thymol serves as a natural active ingredient that aids in the alleviation of associated discomfort (113). Additionally, thymol is used in mouthwash and toothpaste formulations, effectively inhibiting bacterial proliferation in the oral cavity to prevent gingivitis and bad breath (114). In addition, the anti-inflammatory and analgesic properties of thymol have been well-documented. It is frequently employed in the clinical management of respiratory tract disorders, such as pharyngitis and bronchitis, facilitating the alleviation of sore throat and mitigates inflammatory responses (115). It can be incorporated into medicated inhalers or throat lozenges, providing soothing relief and enhancing the overall treatment experience for patients (116, 117). On account of its natural origin and minimal side effects, thymol is also recommended for vulnerable populations such as children and the elderly, who require high safety standards in medications (118). Moreover, the adjuvant role of thymol in cancer therapy has garnered increasing attention. Studies suggest that thymol may regulate cell proliferation and apoptosis, exhibiting inhibitory effects on certain types of tumor cells, thus offering new directions for drug development (79).

Thymol is also extensively utilized in skincare products. With the growing consumer demand for natural and multifunctional skincare products in modern markets, thymol is integrated into formulations to prevent microbial contamination and provide antioxidant protection for the skin. Specifically, thymol is present in a variety of premium skincare products, such as creams, lotions, and toners, where it contributes to retarding skin aging processes and enhancing skin radiance (113, 119).

#### 7.2.2 Joint activity with other substances

Thymol has demonstrated notable synergistic effects when combined with other substances across multiple fields. As structural isomers, thymol and carvacrol possess broad application potential. Their co-supplementation can regulate gut microbiota and treat colitis by activating the cGMP-PKG signaling pathway and inhibiting the mTORC1 pathway, thereby mitigating intestinal inflammation. Additionally, the combination lowers pro-inflammatory cytokine levels while enhancing intestinal barrier functions (120). The application of the thymol-carvacrol combination has also been explored for the treatment of leukemia. Specifically, this combination has been reported to exhibit effective synergistic activity against acute myeloid leukemia (AML) by inducing tumor cell death through multiple pathways. Notably, the combination demonstrates low cytotoxicity toward normal cells, underscoring its therapeutic potential (121). Beyond carvacrol, the thymol-cinnamaldehyde combination has displayed synergistic antibacterial effects (122), with particular efficacy against foodborne pathogens such as *Salmonella*. This combination reduces the required dosage of individual compounds, minimizing potential resistance risks, which makes it suitable for food preservation and clinical anti-infection therapies. Additionally, the application of thymol in conjunction with nanomaterials has attracted increasing attention. Encapsulating thymol within nanoparticles can significantly enhance its bioactivity and stability, expanding its utility in pharmaceutical formulations and cosmetic products (123).

## 8 Conclusion

As a naturally derived monoterpenic phenolic compound, thymol demonstrates substantial application potential and promising prospects in fields such as the food industry, agriculture, and animal husbandry. In recent years, traditional extraction and synthesis methods have gradually been supplemented or replaced by modern green technologies, including ultrasound-assisted extraction, supercritical fluid extraction, and subcritical water extraction. These emerging techniques offer more sustainable and efficient options for the large-scale production of thymol. Notably, Zhu et al. (124) achieved *de novo* biosynthesis of thymol in *Yarrowia lipolytica* for the first time through genetic engineering, providing a new direction for research on thymol biosynthesis. Future studies could focus on improving thymol biosynthetic pathways through advanced gene-editing technologies. Owing to its broad-spectrum antimicrobial, antioxidant, and anti-inflammatory activities, thymol has already been successfully applied to a variety of foods—including meat products, vegetables, and dairy products—primarily as a component of packaging materials or as a food additive to preserve freshness and extend shelf life. Nevertheless, several challenges remain regarding its application in food systems. Future research should emphasize the synergistic combination of thymol with other compounds to reduce its odor and volatility, thereby minimizing negative impacts on food quality while also controlling production costs. Additionally, thymol has been classified as “Generally Recognized as Safe” (GRAS) by the FDA,^7^ and the U.S. EPA^8^ has assigned it a relatively low-risk profile for certain usage. However, data on its pharmacological dosing, therapeutic window, and long-term safety in humans remain insufficient. Therefore, investigations into the toxicological safety of thymol should be prioritized, including assessments of migration levels, intake levels, long-term toxicity, and potential allergic reactions. Such evaluations are essential for regulatory approval and broader commercial application.

In summary, through interdisciplinary collaboration and technological innovation, thymol is poised to play a more significant role in the food industry, emerging as a more natural, safe, and functional bioactive compound.

## Footnotes

1
https://zh.wikipedia.org/wiki

2
https://www.webofscience.com/

3
https://www.cas.org/solutions/cas-scifinder-discovery-platform/cas-scifinder

4
https://www.acs.org/

5
https://www.ncbi.nlm.nih.gov/

6
https://www.megasoftware.net/

7
https://www.fda.gov/

8
https://www.epa.gov/
