# Supplementary figures and images for: Thymol: properties, synthesis, mechanism of action, and applications
Source: Front Nutr. 2026 Mar 20;13:1774718. doi: 10.3389/fnut.2026.1774718 (PMC13047168; doi:10.3389/fnut.2026.1774718)

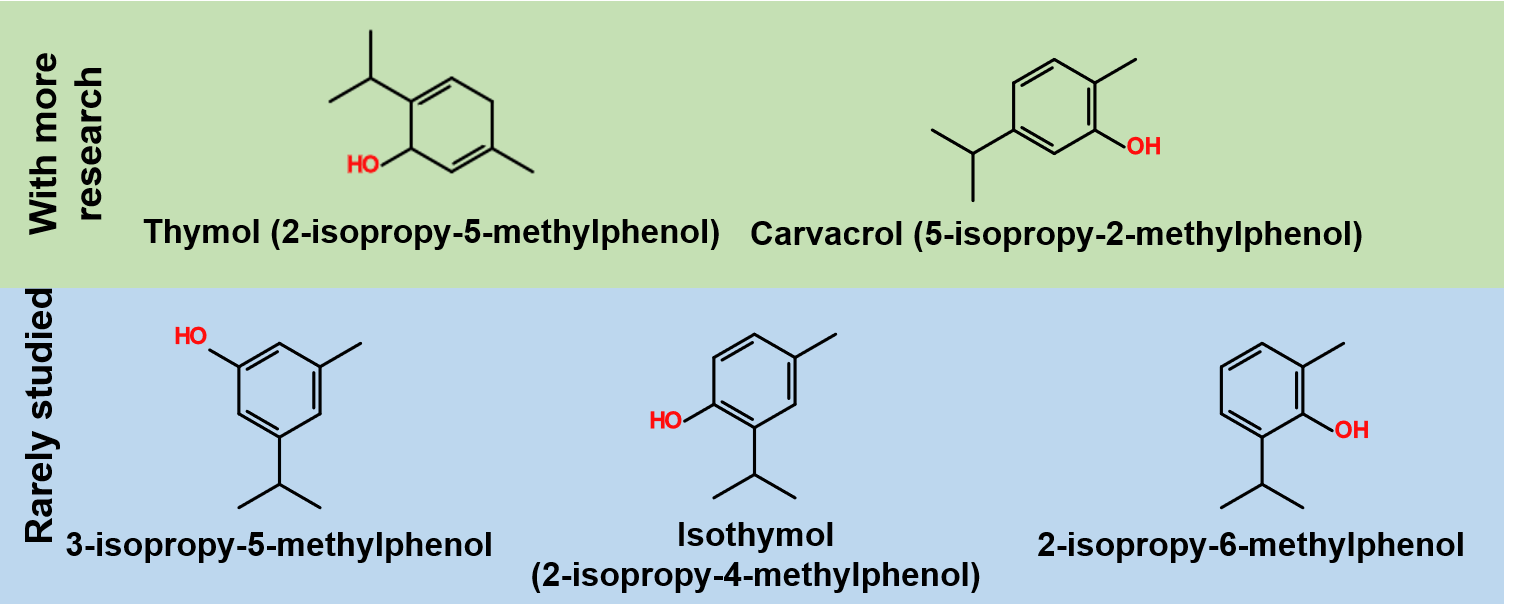

Supplement: Supplementary file 1 [file Image_1.tif]

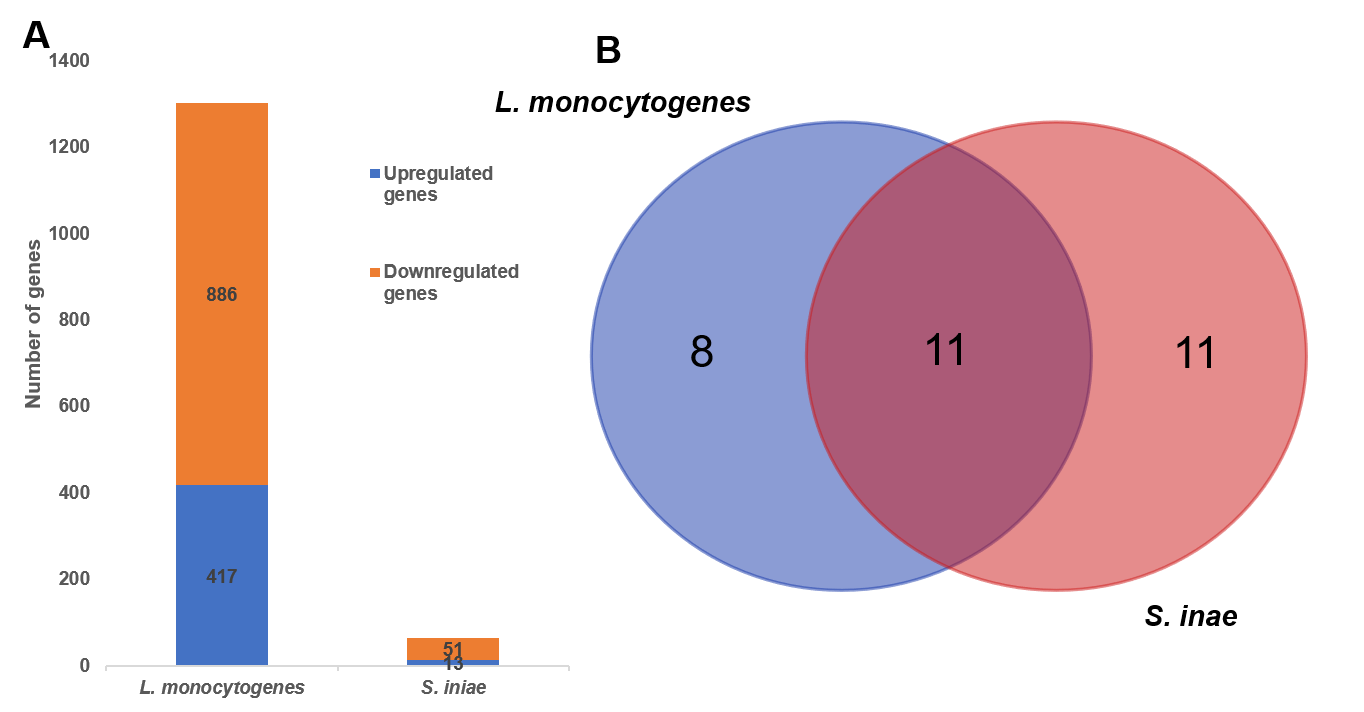

Supplement: Supplementary file 2 [file Image_2.tif]

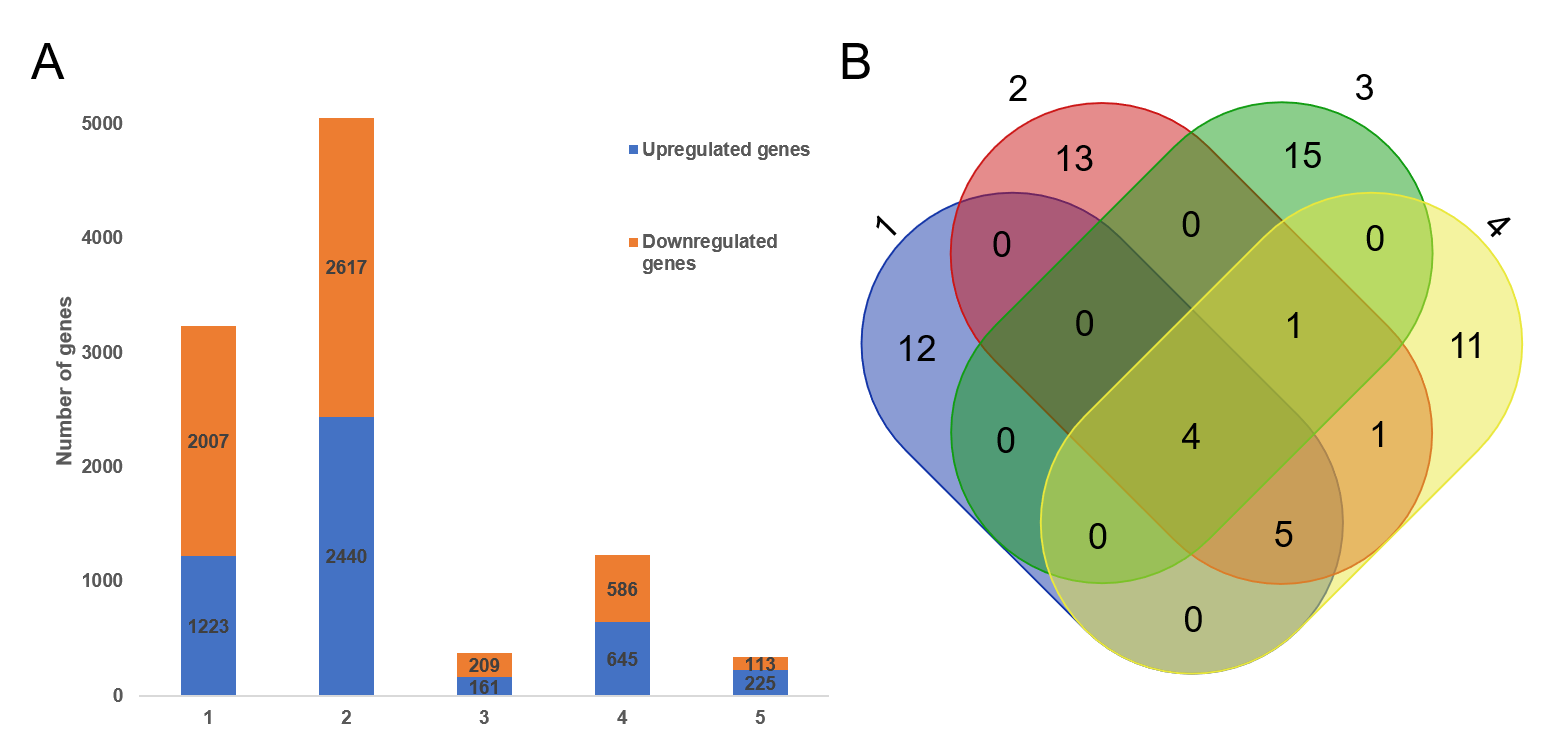

Supplement: Supplementary file 3 [file Image_3.tif]
